# Bacteriological Diphtheria

**Published:** 1902-03-22

**Authors:** J. Odery Symes

**Affiliations:** Assistant Physician and Bacteriologist, Bristol General Hospital


### Hospital Clinics and Medical Progress.

### BACTERIOLOGICAL DIPHTHERIA.

By J. Odery Symes, M.D., D.P.H., Assistant Physician and Bacteriologist, Bristol General Hospital.

The value of a bacteriological examination in suspected cases of diphtheria is so generally acknowledged that the procedure is now widely adopted throughout the country. Many of the larger towns have equipped laboratories in which the specimens are examined free of charge. A large number of sanitary authorities have made arrangements with public laboratories for the examination of cases, and where such facilities are not provided medical practitioners send specimens to be examined at their own cost, so that at the present time probably more than one-half of all cases notified as diphtheria in England have been confirmed by bacteriological diagnosis. That bacteriological methods of diagnosis are of great value to the practitioner there can be no doubt, for by this method alone is it possible in certain cases to distinguish between tonsillitis and true diphtheria. Properly interpreted, bacteriological reports will lead to a marked decrease in the spread of the disease and a diminution in its mortality. At the same time there is a danger, and a very real danger, that the reports, if improperly understood, may lead to exaggerated views as to the incidence of the disease, and, by giving rise to erroneous diagnosis, expose the public to serious risk and unnecessary danger. This is particularly the case where, during an epidemic in a given locality or school, cultures are made from throats or noses of all persons who have been in immediate contact with undoubted cases of diphtheria. It is a fact noted by many observers that true diphtheria bacilli may be present in the throats or noses of healthy persons attending upon patients suffering from diphtheria. Thus, in the recent epidemic at Cambridge and Chesterton, Cobbett found true Klebs-Löffler bacilli in 19 out of 650 healthy persons. In a still larger percentage of cases is it possible to find short varieties of diphtheria bacilli, or bacteria closely resembling these in morphological characters. Thus, in the above-mentioned collection of cases, 15 per cent. showed the presence of Hoffmann's bacillus, a variety which might easily be confused with the shorter forms of the true diphtheria organism. To quote other instances, Hewlett and Murray found pseudo-diphtheria bacilli in 24 per cent. and true diphtheria bacilli in 15 per cent. of 385 children admitted to the Victoria Hospital for diseases other than diphtheria. Rober found diphtheria bacilli in 2·5 per cent. of 600 healthy persons, and Parke and Beebe 8 cases of virulent bacilli, 24 of non virulent bacilli, and 27 of pseudo-diphtheria bacilli in 250 children. From a number of cultures made from the noses of children attending the Bristol General Hospital for diseases other than diphtheria, I am of opinion that pseudo-diphtheria bacilli are present in fully 50 per cent. of children belonging to the working classes, and in cases of atrophic rhinitis a bacillus, in all morphological characters closely resembling the diphtheria bacillus, is almost invariably to be found. Now it is generally agreed that non-virulent forms of the Klebs-Löffler bacillus may under favourable conditions again become virulent, and there is some evidence to show that pseudo-diphtheria (Hoffmann) bacilli may develop into the true Klebs-Löffler variety. The question then arises, "How are we to deal with persons who, although exhibiting no clinical symptoms of diphtheria, yet carry the germs of the disease in the nose or throat?" The bacteriological report simply states that diphtheria bacilli are present or that "short diphtheria forms," or "doubtful," or "suspicious" forms have been detected. To test these forms by inoculation experiments would involve a considerable expenditure of time and money, and although the bacteriologist by various methods of culture and by differential staining processes attempts to pick out the true from the false varieties, yet such attempts can only be partially successful. The final diagnosis must rest upon the medical attendant. If there are marked clinical symptoms of diphtheria, then the condition should be notified and treated as such, whether the bacteriological examination be confirmatory or otherwise. A negative bacteriological result should never be held to contradict definite clinical symptoms until repeated examinations have been made from both nose and throat, and the cultures made on a variety of media. Such procedures must necessarily take several days and it would be unwise to delay the isolation of the patient and the administration of antitoxin until the result was obtained.

Cases in which the clinical symptoms are slight, but possibly suggestive of diphtheria, and in which the bacteriologist reports the finding of "short diphtheria bacilli," "suspicious," or "doubtful" forms should be notified, isolated, and treated as true cases of diphtheria. The most difficult type of case to deal with is that in which there are no clinical symptoms of diphtheria, or only a slight nasal catarrh, such as is extremely common amongst children of the working classes. Cultures taken from such persons may show every grade of bacillus from the long typical Klebs-Löffler forms to the short pseudo-diphtheritic type. It has been customary in some localities to notify such cases as diphtheria, and to send them to isolation hospitals where such exist. By such a course of action the notification returns of diphtheria are swelled, the ratepayers are put to unnecessary expense, and the patient exposed to great personal risk. It has happened that parents have been prosecuted and fined for exposing children who, though apparently in perfect health, yet were ordered to be isolated on account of having "short forms of diphtheria bacilli" in the fauces. Children, also, are sometimes kept at the public expense for weeks in an isolation hospital simply on account of such short forms, and may, and have, during their stay contracted true diphtheria, leading to serious impairment of their general health. From a common-sense point of view it would seem impossible to describe a person as suffering from diphtheria when there are neither objective nor subjective signs or symptoms present, whatever may have been the bacteriological report. The safest course to pursue undoubtedly is to refrain from notifying such cases. Theoretically, persons in apparent health, but carrying diphtheria organisms in their fauces are possible sources of danger to others. It is a matter of great difficulty, however, to directly trace infection to such persons, although strong presumptive evidence has, in many instances, been brought forward. It is desirable, therefore, that this should be explained to the individul or to the friends, and that it should also be pointed out to them that at any time the organisms now non-virulent may awake to activity, and excite an attack of diphtheria. If these facts are clearly laid before the patient's friends, there will rarely be any difficulty in securing a certain degree of precaution, such as the avoidance of close contact with other persons, cessation of school-attendance, and the systematic treatment of the affected parts with suitable antiseptic remedies. Generally far less attention need be given to the results of cultures taken from the nose, than to those from the throat, for in the former situation, bacilli resembling diphtheria bacilli are found in fully 50 per cent. of healthy children. In convalescence from diphtheria it is well known that "short" varieties of the diphtheria bacillus may persist for weeks and months in the upper air-passages. These are seldom virulent, and certainly do not warrant the prolonged detention of the sufferer in hospital as is so often done. A stay in hospital of from a month to six weeks is sufficient as a rule to ensure safety, and nothing so speedily ensures the disappearance of these short varieties as freedom from the confinement of hospital wards. The points we should wish to emphasise are: that the diagnosis of diphtheria cannot be made by the bacteriologist alone; that his report should state clearly whether typical diphtheria bacilli were present or absent, or whether "short," "suspicious" or "doubtful" varieties were found. On the receipt of such a report the medical man must make the diagnosis, notifying only such cases as present clinical symptoms of disease, and warning those who, though infected, are apparently well, that they may themselves contract the disease, and be a possible source of infection to others.

